# Smoking Patterns in Ghanaian Civil Servants: Changes Over Three Decades

**DOI:** 10.3390/ijerph6010200

**Published:** 2009-01-08

**Authors:** Juliet Addo, Liam Smeeth, David A. Leon

**Affiliations:** Department of Epidemiology and Population Health, London School of Hygiene and Tropical Medicine, Keppel Street, London WC1E 7HT, UK E-Mails: liam.smeeth@lshtm.ac.uk (L. S.); dave.leon@lshtm.ac.uk (D. L.)

**Keywords:** Smoking, Ghana, civil servants

## Abstract

The number of smokers in developing countries is expected to increase as markets in high income countries begin to decline and multinational tobacco companies shift their marketing efforts to lower income countries. We determined the prevalence and distribution of smoking in a cross-sectional study of 1,015 urban civil servants in Accra, Ghana (82.7% participation rate) in 2006. The results were compared to the findings from a previous study in 1976 of civil servants in Accra to estimate the changes in smoking patterns over a 30 year period. In our 2006 study, the smoking prevalence rate was 6.1% (95% CI: 4.8–8.9) and 0.3% (95% CI: 0.006–1.4) in men and women respectively. These figures were dramatically lower than the rates of 32% and 5.9% reported for men and women respectively in the previous study. Knowledge of the health risks associated with smoking may have contributed to the lower rates.

## 1. Introduction

Tobacco smoking is among the leading risk factors for death worldwide including low and middle income countries [1]. Smoking alone is estimated to have caused 21% of deaths from cancer worldwide [2]. More than 1 in every 10 cardiovascular deaths in the world in the year 2000 were attributable to smoking, demonstrating that it is an important preventable cause of cardiovascular mortality [3].

There is currently a paucity of epidemiological information on smoking in most of sub Saharan Africa (SSA) where only few population prevalence studies on smoking have been conducted. Available evidence suggests an extensive variation in the reported prevalence of smoking both between and within studies ranging from none in women in Nigeria and South Africa to 77.7% in rural South African men [4,5,6]. Results from the Demographic and Health Surveys of 14 sub Saharan African nations reported smoking prevalence rates ranging from 8.0% to 27.3% in men and negligible use in women in almost all nations [7]. The low reported smoking prevalence from some studies have given an erroneous impression that smoking is not of public health significance in sub Saharan Africa. Consequently, tobacco prevention programs have received little attention in most sub Saharan African countries and only few have developed policies to regulate environmental tobacco smoking, tobacco advertising and marketing, and the use of tobacco in public places. It is however important for countries in sub Saharan Africa to get prepared for an imminent increase in smoking prevalence rates as tobacco markets begin to decline in high income countries. Tobacco companies are likely to intensify efforts at establishing markets in low income countries including countries in sub Saharan Africa. The likely explosion of tobacco consumption, unless prevented, will result in an economic burden of medical and health costs, loss of productivity, loss of foreign exchange if cigarettes are imported, as well as the costs to the individual and their family [8]. A significant burden of cardiovascular disease and cancer from smoking have been reported in black South Africans, even though tobacco consumption has been reported to be lower compared to high income countries [9].

We conducted a survey of civil servants in Accra, Ghana in 2006 to determine the prevalence and distribution of cigarette smoking. We aimed at comparing the findings from this study with information on smoking in civil servants in Accra from an earlier study carried out in 1976 [10] to examine changes that have occurred in the patterns of smoking over the thirty year period.

## 2. Experimental Section

Accra is the capital city of Ghana and is located in the Greater Accra Region. It is entirely urban and its population has increased from 1.5 million in 1984 to 3 million in 2000 [11]. The Civil Service forms part of the Public Service of Ghana. There are currently 26 ministries within the Ghanaian civil service. The Central Administration in Accra, where the study was conducted, has a workforce of 8,317. We randomly selected seven ministries from the list of all central government ministries in Accra, Ghana. All workers in the central administration offices of the seven selected ministries aged 25 years and above were invited to participate in the survey which was conducted between January and March 2006. Pregnant women were excluded from the study. A recruitment system was set up whereby an administrative officer from each sampled ministry or department, was designated a duty to invite all workers to a location within the ministry to participate in the survey. A standard questionnaire to assess the history and current status of cigarette smoking and alcohol consumption (quantity and frequency) was administered to all participants by field workers trained on basic skills of interviewing. The questionnaire was pre-tested at a civil service department which was not included in the final sample. Feedback on the ease of administering the questionnaires as well as the completeness and accuracy of the questions was applied in improving the questionnaire. Study procedures were approved by the ethics committees of the University of Ghana Medical School and the London School of Hygiene and Tropical Medicine and all participants provided written informed consent.

Smoking was categorised according to self-reported cigarette use as: non- smokers if they had never smoked, ex- smokers if they reported previously smoking cigarettes regularly but no longer do, and current smokers if they reported currently smoking cigarettes. The average number of cigarettes smoked in a day was also determined for current smokers. Alcohol consumption was categorised into “never taken alcohol”, “past alcohol consumption” and “current alcohol consumption”. Those currently consuming alcohol were further qualified based on the number of days in a week of alcohol consumption. The participants were assigned to one of four employment grades using the civil service categories. The “Directors” were considered to be the highest grade and included senior administrators and managers; “professional” – comprising of professional staff; “clerical” and the lowest group, the “unskilled” comprising of labourers, cleaners, drivers, catering and security staff. In terms of education, participants were classified as “Primary” where they had completed 9 years of education or less, “Secondary” where they had completed Senior Secondary School comprising 12 years of education; and “Tertiary” where participants had completed university education or higher. All data forms were entered in Excel and were checked for range and internal consistency. Descriptive analysis of data was performed using STATA Release 10 (Stata Corporation, College Station, Texas). Prevalence of smoking was age-standardised to the WHO world standard population [12].

## 3. Results and Discussion

A total of 1,015 out of 1,227 civil servants employed in the seven sampled ministries participated in the study (participation rate 82.7%). Almost 40% of the participants were women. The participants were aged between 25 and 68 years, with a mean age of 44.0 (SD 10.1) years. All employment grades were reasonably well represented. The participants were well educated, with 41.0% having been educated to a tertiary level, compared to 2.8% in the general Ghanaian population [11]. A higher proportion of younger participants i.e. 52% of those aged 35 years or less had completed tertiary education, compared to 27% of those aged 55 years or more.

The age-standardised prevalence of cigarette smoking among the 1,015 participants was 3.9% (95% CI: 3.0–5.6). The prevalence was higher among men, 6.1% (95% CI: 4.8–8.9) compared to 0.3% (95% CI: 0.006–1.4) among women. Among the participants, 48/615 of men with age-standardised prevalence 7.3% (95% CI: 5.8–10.2) and 2/400 of women, prevalence 0.5% (95% CI: 0.06–1.8) were considered to be ex-smokers. The distribution of current and ex-smokers by age, socioeconomic position and alcohol consumption is shown for men and women in Table 1. The prevalence of smoking was highest in men aged 55 years or more and those who were of lower socioeconomic position, characterised by lower education and lower employment grade. Among the participants, 83% of smokers and 49% of non smokers reported consuming alcohol. The prevalence of smoking was highest among men who consumed alcohol frequently (four or more times a week). The prevalence of smoking in men did not differ significantly by marital status or religion. The average number of cigarettes smoked per day among the 41 current male smokers was 4.3 sticks. The average number of cigarettes smoked/day was similar in all age groups; 5 cigarettes/day in men less than 35 years and 4.8 cigarettes per day in those aged 55 years and above. The average number of cigarettes smoked was 4.7 cigarettes per day in unskilled and clerical male workers and 3.7 cigarettes per day among the professionals and directors. The only female who smoked reported smoking an average of 3 sticks of cigarettes per day. About 82% of men reported smoking 5 or less cigarettes in a day while 18% reported smoking 6 to 10 sticks of cigarettes per day. None of the participants smoked more than 10 cigarettes in a day.

A previous study of smoking conducted in Ghanaian civil servants between 1973 and 1976 and which involved 486 males aged 15 to 64 years and 202 females aged 15–54 years, reported a smoking prevalence of 32% for males and 5.9% for females [10]. The proportion of smokers was similar among those who earned low and high salaries. Among smokers, the mean number of cigarettes smoked in a day was 7 for males and 4.7 for females. The civil servants in higher employment grades smoked an average of 12 cigarettes per day while lower salaried workers smoked 7 per day. Among civil servants, 91% of smokers and 60% of non smokers used alcohol. The male participants aged 20–29 years smoked an average of 5.5 cigarettes/day compared to 10 cigarettes/day among those aged 50 to 59 years. Table 2 presents the results on smoking between the two studies of civil servants and the results on smoking in men from the Ghana Demographic and Health Survey conducted in 2003.

### 3.1. Discussion

The prevalence of smoking in our study of civil servants in Accra was low and almost non existent in women. The prevalence of smoking was found to be higher among older men and those of lower socioeconomic position. A greater proportion of men who smoked also consumed alcohol. The majority of the smokers interviewed reported smoking an average of five or less sticks of cigarettes/day. The average number of cigarettes smoked/day did not differ greatly between the older and younger participants but was slightly higher among the participants in the lower employment grades compared to those in the higher grades. The prevalence of smoking in our study and the mean number of cigarettes smoked were markedly lower than that in the previous study conducted among civil servants in Accra three decades ago. The prevalence of smoking was however higher in men compared to women in both studies. The age distribution of smokers differed between the two studies. Whereas the highest prevalence was reported among those aged 20 to 29 years in the previous study, it was highest among those aged 55 years and above in the present study. The prevalence of smoking was similar in civil servants of both high and low socioeconomic status in the previous study, however those of higher socioeconomic status were reported to smoke more heavily. This differed from the findings of the present study where there was a higher prevalence of smoking among those of lower socioeconomic status who also reported smoking slightly more cigarettes per day.

The previous study had described a Ghanaian smoker as an urban male cigarette user starting in adolescence and becoming a heavier smoker in middle age, likely to belong to either lower socioeconomic or higher income group and smoking more heavily if he is in the higher income group. The findings from our present study indicate a slight change in the profile of a smoker. The smoker in the present population of civil servants was a male cigarette user most likely middle aged and of lower socioeconomic status. It could be argued that the higher smoking prevalence in the older ages is an indication of a “cohort effect” and that very few young men are taking up the habit of smoking. The results on smoking from the present study of civil servants are consistent with national Ghanaian data on smoking obtained from the Demographic and Health Survey in 2003 which included over 10,000 individuals aged 15 to 59 across the country. Smoking was found to be rare in women (<1%) and 9% in men. The prevalence of smoking was highest in men aged 35 years or more, those with no education and in the lowest wealth quintile. The mean number of cigarettes smoked by each smoker was 3.7 per day [13]. These findings are consistent with findings from other population surveys from sub Saharan Africa [6,14,15,16]. Estimates from Demographic Health Surveys of men and women in 14 African nations indicate that tobacco smoking in sub Sahara Africa remains low compared to other nations of the world, increasing their vulnerability to further penetration of markets by multinational tobacco companies [7]. It has been suggested that many adverse risk factors such as tobacco use may become more prevalent among poor individuals within poor regions during the 21^st^ century [17]. The low prevalence of smoking and particularly lower prevalence in younger participants and those of higher socioeconomic status in Ghana, are intriguing findings consistent with the later stage of the smoking epidemic in high income countries[18,19,20]. The findings suggest that smoking prevalence in Ghana could possibly have already peaked, declined and become concentrated among those of lower socioeconomic status. Early in the epidemic smoking is typically more common in the higher social classes, but the pattern reverses as people in the higher socioeconomic strata discard the habit in response to health promotion messages with those of lower socioeconomic status taking up these behaviours later [17,18]. Interestingly the decline in smoking prevalence and consumption of cigarettes in Ghana have occurred in the absence of a comprehensive national tobacco control programme. There have been a number of policy proposals to ban smoking in public places and direct advertising of cigarettes and tobacco in Ghana. There is however as yet no legislation around tobacco control and there have been no strong public health measures and interventions undertaken to arrest the growth in tobacco consumption. The generally low smoking prevalence cannot be explained by the cost of cigarettes as the prevalence was higher among those who were of lower socioeconomic status. Social pressures which discourage tobacco use may partly explain the low prevalence of smoking observed in Ghana. Smoking is generally not a culturally desirable behaviour and is particularly unacceptable in women, which is reflected in the very low levels of smoking observed for women.

The present study of smoking in urban Ghanaian civil servants did have some limitations. The sample was not nationally representative and included only urban participants with a higher level of education, and the findings can therefore not be generalised to the entire Ghanaian population. Another concern is the different sampling and data collection methods used in the two civil service surveys. The earlier sample of 20% of all civil servants might differ from the more recent sample of civil servants from seven ministries. The civil servants from the sampled ministries however did not differ significantly by age, sex, level of education and employment grade from workers in other civil service ministries. The low number of smokers in the population limited our ability to perform regression analysis to determine statistical associations between smoking and some exposures in this population. The study nevertheless adds to the limited data on smoking prevalence and patterns in African populations, and allows a comparison of smoking prevalence over a 30 year period.

Despite the current low prevalence of smoking reported in Ghana and some other African nations, it is important for governments of these nations to put in place policies and programs that would control tobacco use and prevent an increase in the prevalence of smokers.

## 4. Conclusions

The tobacco epidemic in Africa can be arrested before it really takes off since an out-of-control tobacco epidemic can clearly not be afforded by the continent [21]. It is important for effective policies on smoking to be implemented urgently and for public education campaigns on smoking to be initiated with particular emphasis on educating men of lower socioeconomic status and encouraging the low smoking prevalence in women.

## Figures and Tables

**Table 1:** Prevalence and distribution of current and ex-smokers in the study population.

	Men			Women		
	Distribution of current smokers (n/N)	Smoking prevalence (95% confidence intervals)^a^	Ex-smoking prevalence (95% confidence intervals)^a^	Distributio n of current smokers (n/N)	Smoking prevalence^b^	Ex-smoking prevalence^b^
**Age groups (years)** 25–34 35–44 45–54 >=55 All ages a	2/125 10/156 18/218 11/116 41/615	1.6 (0.2 –5.7) 6.4 (3.1–11.5) 8.3 (5.0–12.7) 9.5 (4.8–16.3) 6.1 (4.8–8.9)	7.2 (3.3–13.2) 5.8 (2.7–10.7) 10.6 (6.8–15.4) 6.0 (2.5–12.0) 7.3 (5.8–10.2)	0/107 1/93 0/140 0/60 1/400	0 1.1 0 0 0.3	0 0 0.7 1.7 0.5
**Level of education** Primary Secondary Tertiary	22/189 8/153 11/269	9.7 (7.4 –17.1) 4.5 (2.3–10.07) 3.6 (2.1–7.2)	9.1 (6.6–15.9) 5.1 (2.3–10.0) 6.9 (4.6–11.2)	0/88 0/161 0.1/147	0 0 0.7	2.27 0 0
**Civil service employment grade** Unskilled Clerical Professional Director	23/164 2/105 15/253 1/93	11.9 (9.1–20.3) 1.5 (0.2–6.7)5.7 (3.4–9.6) 0.6 (0.03–5.8)	9.9 (6.2–16.1) 5.8 (3.3 –14.5) 7.7 (4.9–11.9) 3.8 (0.7–9.1)	0/47 0/200 1/127 0/26	0 0 0.8 0	2.1 0 0 0
**Alcohol use** Never Stopped Once/week 2–3 times/week 4 or more/week	8/244 2/42 11/173 5/88 15/68	3.3 (1.4–6.4) 4.8 (0.6–16.1) 5.4 (3.2–11.1) 4.5 (1.9–12.8) 22.1 (12.9–33.8)	2.7 (0.9–5.3) 22.6 (13.9–42.0) 5.2 (2.8–10.4) 11.2 (4.0–17.1) 17.5 (10.6–30.5)	0/220 0/21 1/140 0/11 0/8)	0 0 0.7 0 0	0.5 0 0.7 0 0
**Marital status** Single Married Divorced Widowed	3/100 34/487 2/18 2/4	6.0 (0.6–8.5) 5.7 (4.9–9.6)5.4 (1.4–34.7) 14.5 (6.8–93.2)	2.3 (2.2–12.6) 6.8 (5.4–10.2) 13.6 (9.7–53.5) 0	0/102 0/227 1/45 0/26	0 0 2.2 0	0 0.9 0 0
**Religion** Christian Muslim Other	37/541 3/48 1/20	6.1 (4.9–9.3) 6.5 (1.3–17.2) 4.3 (0.1–24.9)	7.3 (5.8–10.5) 6.9 (1.3–17.2) 8.8 (0.1–24.9)	0/386 1/6 0/5	0 16.7 0	0.5 0 0

^a^ prevalence age standardised to the WHO world standard population;

^b^ confidence interval not determined for women because of the low prevalence of smoking.

**Table 2:** Study and smoking characteristics compared between two studies of civil servants in Accra and the Ghana Demographic and Health Survey 2003.

	Study between 1973 and 1976 (Pobee*et al*.)[10]	Study conducted in 2003 (Ghana Demographic and Health Survey) [13]	Study conducted in 2006
**Sample size**	688	5015	1015
**Setting**	Accra	Ghana	Accra
**Participants**	20% of all civil servants	Nationally representative sample of men aged 15–59 years from 6251 households throughout Ghana	All civil servants from 7 randomly sampled ministries
**Prevalence of smoking**	32% in men and 5.9% in women	9% in men	6.7% in men and 0.3% in women*
**Mean number of cigarettes/day**	7 in men and 4.7 in women	3.7 per day with 78% smoking 1–5 cigarettes per day	4.3 in men and 3 in women*
**Age and smoking**	Highest prevalence in 20–29 year group. Mean number of cigarettes/day lower in 20 –29 compared to 50 –59 year olds	Highest prevalence in the oldest age group (35 years and above)	Highest prevalence in >=55 years. Mean number smoked/day similar in those less than 35 and those >=55 years old
**Socioeconomic status and smoking**	Similar prevalence in high and low earners. Mean number of cigarettes/day higher in professionals compared to lower salaried workers.	Higher prevalence in rural (10.8%) compared to urban areas (6.8%). Higher prevalence in those with no education and those in the lowest wealth quintile	Higher prevalence in lower socioeconomic group. Mean number of cigarettes/day higher in lower grades of employment compared to higher

^*^only one woman smoked.
